# Supplementary material for: Polarisation of decayless kink oscillations of solar coronal loops
Source: Nat Commun. 2023 Sep 12;14:5298. doi: 10.1038/s41467-023-41029-8 (PMC10497544; doi:10.1038/s41467-023-41029-8)
Supplement: Supplementary file 2 — Description of additional supplementary files [file 41467_2023_41029_MOESM2_ESM.pdf]

Description of Additional Supplementary Files

File Name: Supplementary Movie 1
Description: The oscillating coronal loop bundle observed by the HRIEUV 174 Å (a) and AIA 171 Å images (original in the b and magnified by a factor of 5 in the c.

File Name: Supplementary Movie 2
Description: The simulated oscillating loop with different types of polarisation, seen in two LoS similar to the observations.
